# Altered Natural Killer Cell Function in HIV-Exposed Uninfected Infants

**DOI:** 10.3389/fimmu.2017.00470

**Published:** 2017-04-24

**Authors:** Christiana Smith, Emilie Jalbert, Volia de Almeida, Jennifer Canniff, Laurel L. Lenz, Marisa M. Mussi-Pinhata, Rachel A. Cohen, Qilu Yu, Fabiana R. Amaral, Jorge Pinto, Jorge O. Alarcon, George Siberry, Adriana Weinberg

**Affiliations:** ^1^Department of Pediatric Infectious Diseases, University of Colorado School of Medicine, Aurora, CO, USA; ^2^Federal University of Sao Carlos Biological and Health Sciences Center, Sao Carlos, Brazil; ^3^Department of Immunology and Microbiology, University of Colorado School of Medicine, Aurora, CO, USA; ^4^Department of Pediatrics, Ribeirão Preto School of Medicine, University of São Paulo, São Paulo, Brazil; ^5^Westat, Rockville, MD, USA; ^6^Federal University of Minas Gerais, Belo Horizonte, Brazil; ^7^Instituto de Medicina Tropical “Daniel A. Carrión” de la Universidad Nacional Mayor de San Marcos, Lima, Perú; ^8^Maternal and Pediatric Infectious Disease Branch, Eunice Kennedy Shriver National Institute of Child Health and Human Development, Bethesda, MD, USA

**Keywords:** HIV-1, infant, natural killer cells, K562 cells, interleukin-12, respiratory tract infections

## Abstract

**Objectives:**

HIV-exposed uninfected (HEU) infants have higher rates of severe and fatal infections compared with HIV-unexposed (HUU) infants, likely due to immune perturbations. We hypothesized that alterations in natural killer (NK) cell activity might occur in HEU infants and predispose them to severe infections.

**Design:**

Case–control study using cryopreserved peripheral blood mononuclear cells (PBMCs) at birth and 6 months from HEU infants enrolled from 2002 to 2009 and HUU infants enrolled from 2011 to 2013.

**Methods:**

NK cell phenotype and function were assessed by flow cytometry after 20-h incubation with and without K562 cells.

**Results:**

The proportion of NK cells among PBMCs was lower at birth in 12 HEU vs. 22 HUU (1.68 vs. 10.30%, *p* < 0.0001) and at 6 months in 52 HEU vs. 72 HUU (3.09 vs. 4.65%, *p* = 0.0005). At birth, HEU NK cells demonstrated increased killing of K562 target cells (*p* < 0.0001) and increased expression of CD107a (21.65 vs. 12.70%, *p* = 0.047), but these differences resolved by 6 months. Stimulated HEU NK cells produced less interferon (IFN)γ at birth (0.77 vs. 2.64%, *p* = 0.008) and at 6 months (4.12 vs. 8.39%, *p* = 0.001), and showed reduced perforin staining at 6 months (66.95 vs. 77.30%, *p* = 0.0008). Analysis of cell culture supernatants indicated that lower NK cell activity in HEU was associated with reduced interleukin (IL)-12, IL-15, and IL-18. Addition of recombinant human IL-12 to stimulated HEU PBMCs restored IFNγ production to that seen in stimulated HUU cultures.

**Conclusion:**

NK cell proportion, phenotype, and function are altered in HEU infants. NK cell cytotoxicity and degranulation are increased in HEU at birth, but HEU NK cells have reduced IFNγ and perforin production, suggesting an adequate initial response, but decreased functional reserve. NK cell function improved with addition of exogenous IL-12, implicating impaired production of IL-12 by accessory cells. Alterations in NK cell and accessory cell function may contribute to the increased susceptibility to infection in HEU infants.

## Introduction

HIV-exposed uninfected (HEU) infants represent a large and growing population with unique health-care needs. Compared with HIV-unexposed uninfected (HUU) infants, HEU infants are at increased risk of morbidity and mortality from lower respiratory tract infections (LRTI), sepsis, and gastrointestinal infections (1–5). The pathogens responsible for infections in HEU infants include respiratory syncytial virus (RSV) and parainfluenza viruses (6, 7), as well as encapsulated bacteria such as Pneumococcus and group B *Streptococcus* (8–10). HEU infants also have impaired vaccine responses, including decreased T-cell responses to the BCG vaccine (11–14), decreased cellular and humoral immune responses to the tetanus vaccine (14, 15), and low antibody titers and avidity in response to the measles and *Haemophilus influenzae* type b vaccines (16, 17). The risk of infection in HEU infants is inversely correlated with maternal CD4 cell count, suggesting that inflammation associated with maternal HIV infection may adversely impact fetal immune development (3, 10, 18). HEU infants acquire lower levels of maternal antibodies during gestation (19–21) but also demonstrate numerous perturbations of their own immune system. These perturbations likely contribute to the increased susceptibility to infection and decreased vaccine responses in HEU infants.

Few studies have examined innate immune responses in HEU infants. Natural killer (NK) cells are innate lymphocytes that play an important role in the control of viral infections, especially in early life while the adaptive immune response is immature (22, 23). NK cells also act both as activators and effectors of the adaptive immune response (24). NK cells can be divided into the CD16^+^CD56^dim^ cells, which release cytotoxic granules such as perforin upon interaction with target cells, and the CD16^−^CD56^bright^ cells, which produce cytokines such as interferon (IFN)γ when stimulated (25). In a small study of Kenyan HEU infants, NK cells showed increased markers of activation and decreased perforin expression compared to HUU infants (26). In addition, two studies comparing NK cells from HEU and HIV-infected infants found a more activated phenotype of killer immunoglobulin-like receptors in HEU infants (27, 28).

We hypothesized that alterations in NK cell phenotype and function in HEU infants might contribute to their increased risk of infection in early life. We tested this hypothesis using samples from infants enrolled in the NICHD International Site Development Initiative Longitudinal Study in Latin American Countries cohort. Twenty percent of HEU infants in this cohort experienced LRTI in the first 6 months of life, nearly half of whom required hospitalization (5, 29). We found differences in NK cell phenotype and function between HEU and HUU infants from similar geographic locations and examined the relationship between NK cell characteristics in HEU infants and their risk of developing LRTI in the first 6 months of life.

## Materials and Methods

### Participants and Specimen Collection

HIV-infected mothers were enrolled from 2002 to 2009 and HIV-uninfected mothers were enrolled from 2011 to 2013, as previously described (30). Inclusion criteria for HEU and HUU infants included term gestation (≥37 weeks), singleton, birth weight ≥2,500 g, no congenital anomalies, and follow-up until 6 months of life. All HIV-infected mothers received antiretroviral treatment; 48% received a three-drug combination. All HEU infants received zidovudine prophylaxis and were fed formula. All HEU infants were HIV-uninfected, as defined by ≥2 negative HIV nucleic acid tests (≥1 and ≥4 months of age), or ≥2 negative HIV-1 antibody tests (at least 1 ≥6 months of age). To ensure maximum comparability with HEU infants, we targeted HUU infants with minimal breastfeeding using two-step enrollment. First, we enrolled HUU infants at delivery who met the standard inclusion criteria. At 4–6 weeks postpartum, mothers were contacted by telephone and infants who were fed 0–50% breast milk were invited to continue in the study (Figures S1 and S2 in Supplementary Material). Clinical data were collected for each infant, including the incidence of LRTI in the first 6 months of life.

Most HEU samples were obtained from infants enrolled in 11 sites in Brazil, except 5 HEU samples used for the interleukin (IL)-12 reconstitution experiments, which were obtained from a single site in Peru. All HUU samples were obtained from infants enrolled in a single site in Brazil. Peripheral venous blood was collected from infants at birth before hospital discharge and at 6 months of age. Peripheral blood mononuclear cells (PBMCs) were isolated on-site by Ficoll-Hypaque gradient centrifugation, cryopreserved, and stored at −150°C or in liquid nitrogen until needed (31, 32).

### NK Cell Phenotyping and Functional Assays

After thawing, PBMCs were washed twice with RPMI 1640 containing l-glutamine (Gibco) with 10% fetal bovine serum (SAFC Biosciences) and 2 µL/mL Benzonase (Novagen), and then resuspended in RPMI 1640 containing l-glutamine supplemented with 10% human AB serum (GemCell) and 1% penicillin–streptomycin (Gibco) (complete media). Cell counts and viability were obtained using a guava easyCyte™ instrument (Millipore) and samples with a minimum of 40% viability and 800,000 viable cells were included. PBMCs were rested in complete media at 37°C in a humidified 5% CO_2_ incubator for 4 h prior to stimulation.

K562 human myelogenous leukemia cells (ATCC #CCL-243) were cultured in complete media at 37°C, 5% CO_2_. K562 target cells were harvested in the log phase of growth and labeled with carboxyfluorescein succinimidyl ester (CFSE; Life Technologies; final concentration of 0.03 μL/10^6^ cells) to discriminate from effector cells. PBMCs were incubated with CFSE-labeled K562 cells at an effector-to-target ratio of 5:1 in 96-well plates. For experiments using IL-12, recombinant human (rh)IL-12p70 (BioLegend) was added to the cell culture at the manufacturer-recommended concentration of 0.1 ng/mL. Unstimulated PBMCs were incubated for each subject as a negative control. In each experiment, PBMCs from a healthy adult donor were used as a positive control, and K562 cells were incubated without effector cells as a marker of spontaneous cell death. Anti-CD107a Brilliant Violet 421 monoclonal antibody (mAb; BD Biosciences) was added at the beginning of the stimulation period. Cell cultures were incubated for 20 h at 37°C, 5% CO_2_; the secretion inhibitors Brefeldin A and Monensin (Sigma) were added at a final concentration of 5 µg/mL each during the final 4 h of stimulation.

After incubation, PBMCs and K562 cells were stained with the live/dead discriminator Zombie Yellow (ZY; Biolegend), and mAbs against the following: CD3 APC-Cy7, CD14 APC-Cy7, CD19 APC-Cy7, CD16 PerCP-Cy5.5, CD56 Alexa700 (BD Biosciences), and NKG2C PE (R&D Systems). Cells were fixed with FACS lysing solution and permeabilized with FACS permeabilizing solution (BD) and stained for intracellular markers with anti-IFNγ PE-Cy7 (BD Biosciences) and anti-perforin APC (Biolegend) mAbs. Sample acquisition was performed on a 10-channel Gallios™ flow cytometer (Beckman Coulter) and analyzed using FlowJo version 9.9.5b1 software (FlowJo LLC, Ashland, OR, USA).

**Figure 1:**
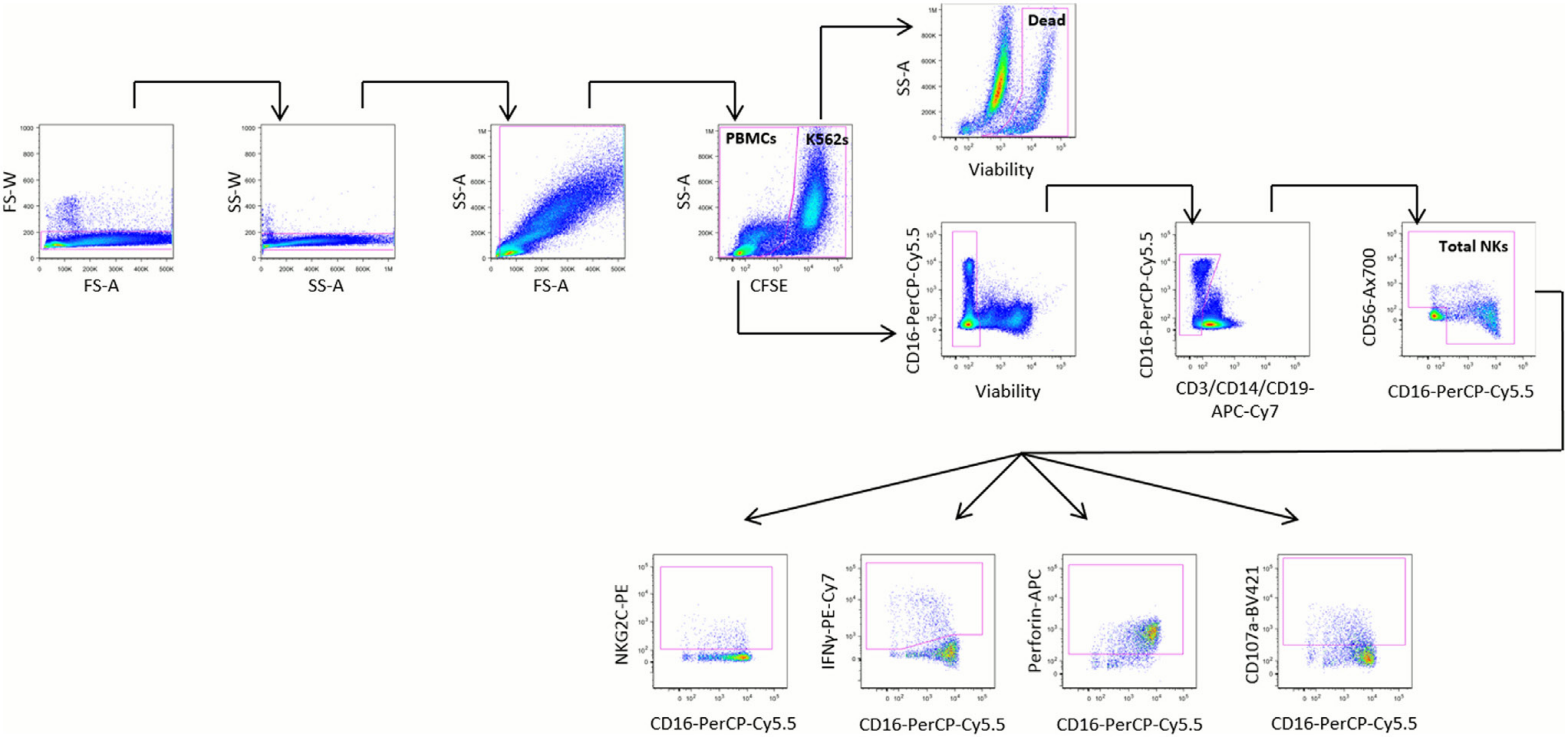
**Representative gating strategy**. Peripheral blood mononuclear cells (PBMCs) were stimulated for 20 h at a 5:1 ratio with K562 cells and stained with the monoclonal antibodies shown. PBMCs were identified by sequentially gating out doublets, debris, and carboxyfluorescein succinimidyl ester (CFSE)-stained K562 cells. Dead K562 cells were defined as CFSE^+^ZY^+^ cells. Natural killer (NK) cells were identified from PBMCs by gating out non-viable cells, then gating on cells negative for CD3, CD14, and CD19 and positive for CD16 and/or CD56. The relative proportions of NK cells expressing NKG2C, interferon (IFN)γ, perforin, and CD107a were identified among all NK cells, CD16^−^ NK cells, and CD16^+^ NK cells.

Natural killer cells were defined as negative for the lineage markers CD3, CD14, and CD19 and positive for CD16 and/or CD56 (Figure 1). Cell marker data are described as the percentage of stimulated NK cells that expressed the marker, or as mean fluorescence intensity (MFI). Upregulation of cell markers is reported as the percentage of unstimulated NK cells subtracted from the percentage of stimulated NK cells expressing the marker of interest. Target cell death was calculated using the following equation, where dead target cells are defined as CFSE^+^ZY^+^:

$$\begin{array}{l} \%\text{target cell death}=\left[\frac{\%\text{dead targets}-\%\text{spontaneous dead targets}}{100-\%\text{spontaneous dead targets}}\right]\times 100 \end{array}$$

As part of the validation of our assay, we evaluated the effect of PBMC viability on the proportion of NK cells among PBMCs, and on the expression of IFNγ by NK cells, and found no correlation between viability and either of these parameters (Figure S3 in Supplementary Material).

### Analysis of Cell Culture Supernatant

Biomarkers of immune activation [IFNα, IFNγ, IL-2, IL-10, IL-12, IL-15, IL-18, macrophage inflammatory protein (MIP)-1α, MIP1β, and tumor necrosis factor (TNF)-α] were measured in cell culture supernatant using electrochemiluminescent bridging immunoassay technology (Meso QuickPlex SQ 120, MesoScale Discovery, Gaithersburg, MD, USA). Expression of each biomarker is reported as the median fold increase in analyte detection between cell culture supernatants of unstimulated and K562-stimulated PBMCs.

### Statistical Analyses

Statistical analyses were performed using Prism software version 6 (GraphPad, La Jolla, CA, USA). All tests were two tailed. Measures of central tendency are expressed as median with interquartile range. The between-group differences were compared by Mann–Whitney tests with statistical significance defined as *p* < 0.05.

## Results

### Study Population

Baseline characteristics of mothers and infants are reported in Table 1. Many characteristics, such as geographic location, maternal age, and number of people in the household (which can be an important indicator of the burden of pathogens to which an infant is exposed), were not significantly different between HEU and HUU groups. Other characteristics, such as gestational age and birth weight, were significantly different, but these differences of 3 days in gestational age and of <10% in birth weight were not deemed biologically significant. Maternal education and employment were higher in the HUU infant group; these demographics are characteristic of the HIV epidemic in Brazil and were difficult to control for.

**Table 1:** **Demographic characteristics of mothers and infants according to infant HIV exposure category**.

	Infant HIV exposure category		*p*-Value^b^
	HEU (*N* = 60)	HUU (*N* = 72^a^)	
**Maternal characteristics**			
Mean (SD) maternal age at enrollment	28.0 (5.1)	25.9 (6.2)	0.05
**Substance use during pregnancy, *N* (%)**			
Alcohol	13 (21.7)	8 (11.1)	0.15
Tobacco	16 (26.7)	10 (13.9)	0.08
Marijuana	4 (6.7)	0 (0.0)	0.04
Crack or cocaine	2 (3.3)	0 (0.0)	0.20
Maternal employment, *N* (%)	22 (36.7)	41 (56.9)	0.02
Mean (SD) years of completed education	7.6 (3.2)	9.3 (2.4)	0.001
Mean (SD) number of prior pregnancies	2.3 (1.9)	1.2 (1.3)	<0.0001
Mean (SD) parity	1.8 (1.6)	1.0 (1.1)	0.001
Mean (SD) number of people living in the household	4.4 (2.0)	4.7 (1.7)	0.32
Mean (SD) CD4 count (cells/mm^3^) at hospital discharge	563.9 (266.7)	–	–
Mean (SD) CD4% at hospital discharge	32.0 (9.4)	–	–
Mean (SD) viral load (log_10_ copies/mL) at hospital discharge	2.23 (0.81)	–	–
Viral load <400 copies/mL at hospital discharge, *N* (%)	51 (85.0)	–	–
ARV use during pregnancy, *N* (%)		–	–
Zidovudine monotherapy	25 (41.7)	–	–
Combination antiretrovirals	35 (58.3)		
**Infant characteristics**			
Mean (SD) gestational age at birth (weeks)	38.7 (1.2)	39.2 (1.4)	0.01
Female gender, *N* (%)	25 (41.8)	36 (50.0)	0.38
Mean (SD) birth weight (g)	3176.4 (381.5)	3346.5 (422.7)	0.02
Small for gestational age (<10th percentile), *N* (%)	2 (3.3)	0 (0.0)	0.20
Large for gestational age (>90th percentile), *N* (%)	7 (11.7)	16 (22.2)	0.17
Not breastfeeding, *N* (%)	60 (100)	1 (1.4)	<0.0001

*^a^Demographics were missing for 17 HUU subjects. Demographic characteristics for the entire cohorts from which these subjects were selected are presented in Table S1 in Supplementary Material*.

*^b^All p values were calculated by two-sample t-test or chi-square/Fischer’s exact test, where appropriate*.

*Maternal race was not analyzed due to the inability to establish it in this highly mixed Brazilian population*.

*ARV, antiretroviral; HEU, HIV-exposed uninfected infant; HUU, HIV-unexposed uninfected infant; N, number*.

For NK cell phenotyping and functional assays, a subset of samples that contained a minimum of 800,000 viable PBMCs were selected from the infant subjects who met inclusion criteria. Twelve HEU and 22 HUU samples were studied at birth, and 52 HEU and 71 HUU samples were studied at 6 months. The infants studied at birth and 6 months were distinct, with the exception of 4 HEU infants and 4 HUU infants who were studied at both time points. Six additional HEU samples from 6 months of age were randomly chosen to study the effect of IL-12 on NK cell responses.

### Proportion of NK Cells in HEU and HUU Infants

**Figure 2:**
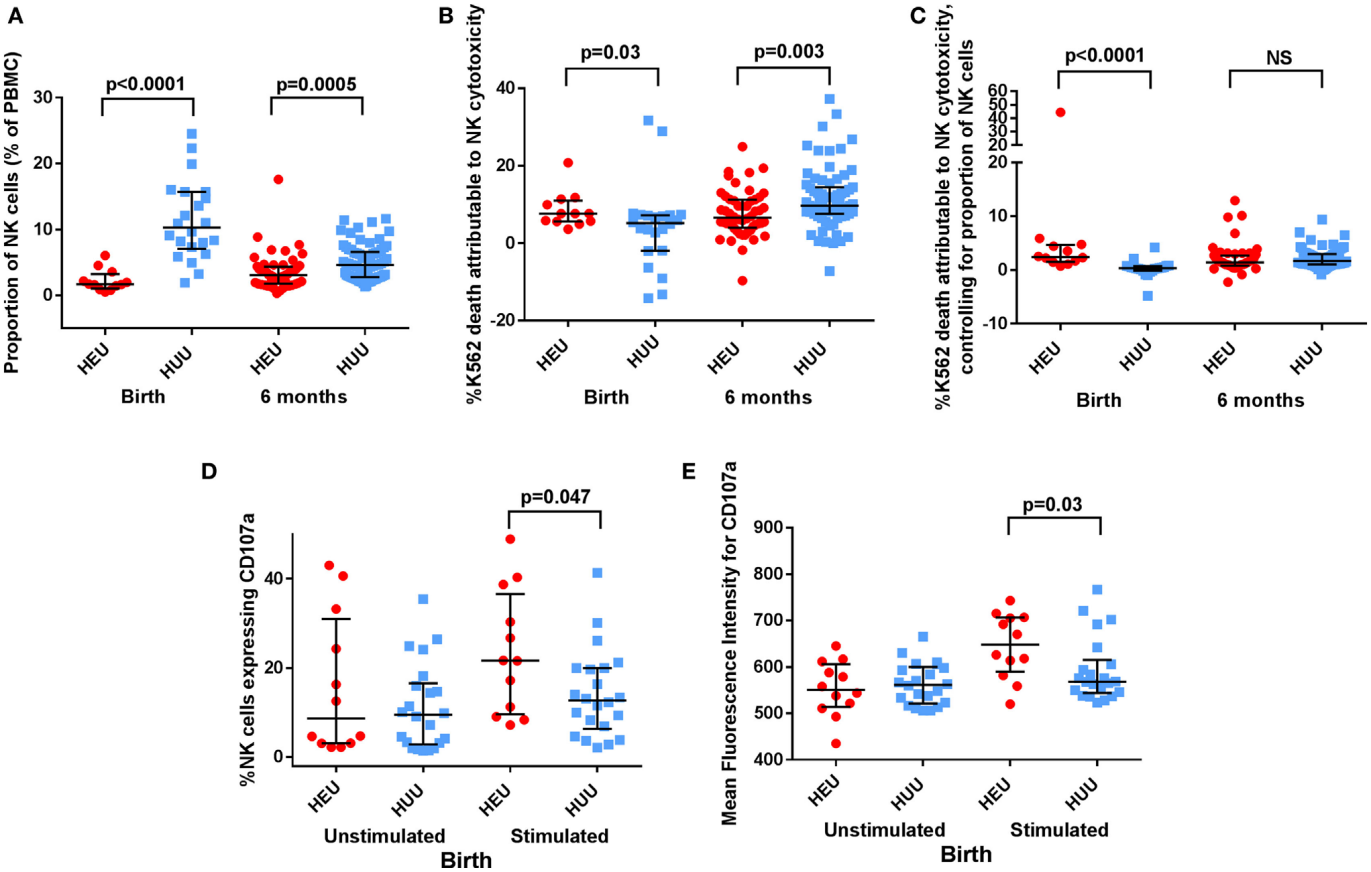
**Proportion, killing, and degranulation of natural killer (NK) cells**. **(A)** The proportion of NK cells among peripheral blood mononuclear cells (PBMCs) is compared between HIV-exposed uninfected (HEU) and HIV-unexposed uninfected (HUU) infants at birth and 6 months. **(B)** The proportion of K562 death attributable to NK cytotoxicity is compared between HEU and HUU infants at birth and 6 months. **(C)** The proportion of K562 death attributable to NK cytotoxicity after controlling for proportion of NK cells, as defined by proportion of K562 death attributable to NK cytotoxicity/proportion of NK cells, is compared between HEU and HUU infants at birth and 6 months. **(D)** CD107a expression, shown as the proportion of NK cells that express CD107a and **(E)** as the mean fluorescence intensity of CD107a^+^ NK cells, is compared between HEU and HUU infants at birth. Mann–Whitney tests were used to compare groups, and *p* values are shown where *p* < 0.05. NS, not significant. Error bars indicate median and interquartile range.

The proportion of NK cells (CD16^+^ and/or CD56^+^; see Figure 1) among PBMCs was lower in HEU compared with HUU infants at birth (*p* < 0.0001) and 6 months (*p* = 0.0005) (Figure 2A). Excluding the 8 infants who were studied at both time points, HEU infants showed a trend toward higher NK cell proportions at 6 months compared to birth (increase from median of 1.85% at birth to 3.12% at 6 months; *p* = 0.13), whereas HUU infants had lower NK cell proportions at 6 months compared to birth (decrease from 9.4% at birth to 4.65% at 6 months; *p* < 0.0001).

### Killing of Target Cells and Degranulation by NK Cells of HEU and HUU Infants

At birth, K562 target cell death attributed to NK cytotoxicity was greater in HEU cultures compared with HUU cultures [7.64% (5.58–11.35) vs. 5.16% (−1.89 to 7.22); *p* = 0.03 (Figure 2B)]. By contrast at 6 months, K562 killing was reduced in HEU cultures compared with HUU cultures [6.56% (5.10–9.12) vs. 9.69% (8.78–11.95); *p* = 0.003]. After adjusting for the proportion of NK cells in each cell culture, the K562 killing attributed to NK cytotoxicity at birth was increased in HEU vs. HUU NK cells (*p* < 0.0001) but did not differ between groups at 6 months (Figure 2C).

CD107a is a marker of NK cell degranulation. Following K562 stimulation, we observed that an increased proportion of birth HEU NK cells stained positive for CD107a compared with HUU [21.65% (9.06–38.70) vs. 12.70% (6.93–20.0); *p* = 0.047 (Figure 2D)]. The intensity of CD107a staining (MFI) was also increased in the birth HEU compared to HUU NK cells [648 (582–707) vs. 569 (546–606); *p* = 0.03 (Figure 2E)]. The increased CD107a expression was only seen in the CD16^+^ NK cell subset. There were no differences between HEU and HUU infants in the proportion or MFI of CD107a^+^ NK cells at 6 months. There were also no differences between HEU and HUU infants in the proportion or MFI of NKG2C^+^ NK cells at either birth or 6 months. Taken together, the increased cytotoxicity and increased CD107a staining of birth HEU NK cells suggests that NK cells from newborn HEU infants have an increased capacity for cytotoxicity. However, by 6 months of age, the cytotoxicity of HEU and HUU NK cells is more similar.

### IFNγ and Perforin Production by NK Cells of HEU and HUU Infants

**Figure 3:**
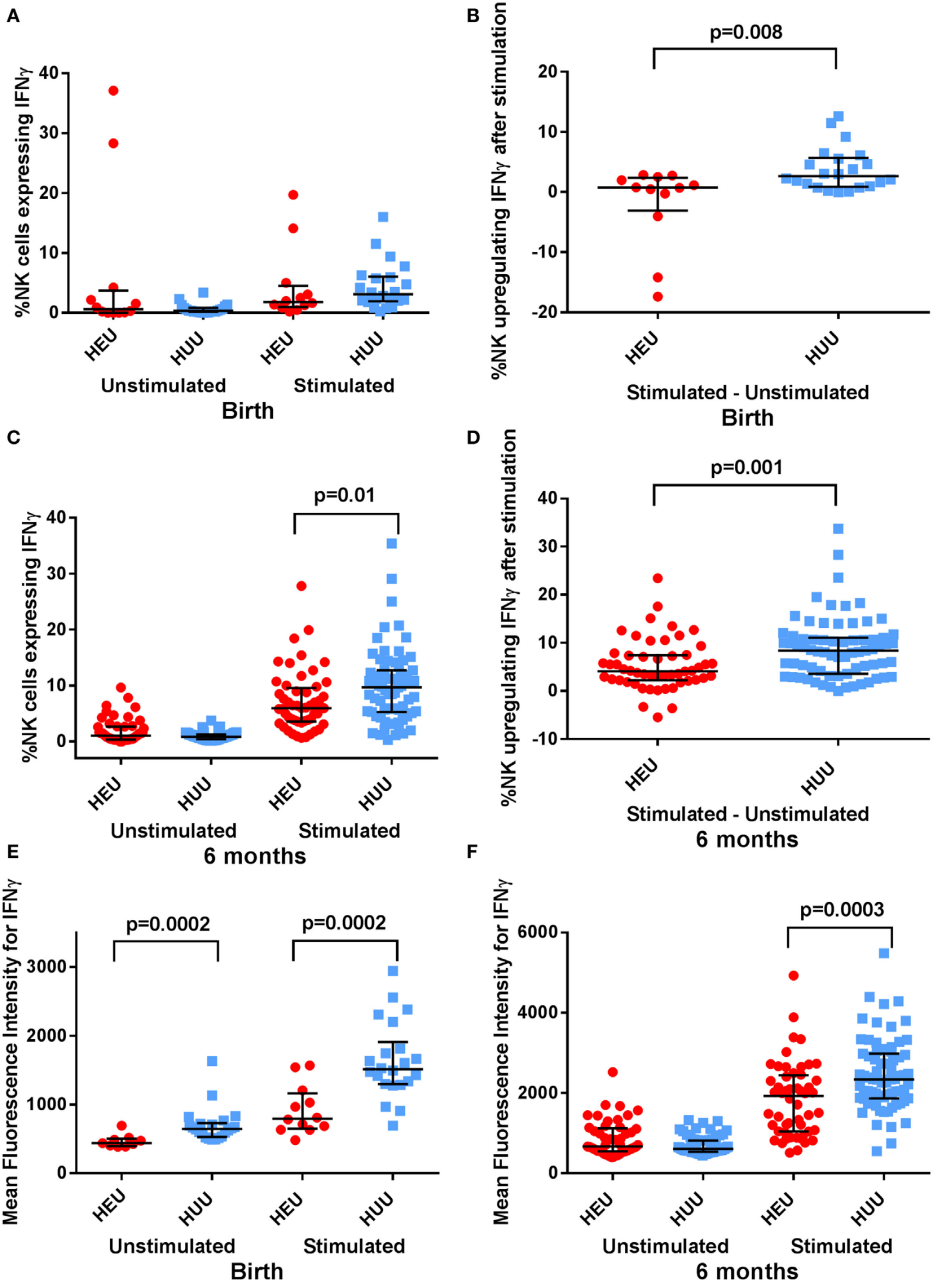
**Interferon (IFN)γ expression by natural killer (NK) cells**. **(A–D)** The proportion of NK cells expressing IFNγ, and the proportion of NK cells upregulating IFNγ (defined by proportion of stimulated NK cells expressing IFNγ—proportion of unstimulated NK cells expressing IFNγ) is compared between HIV-exposed uninfected (HEU) and HIV-unexposed uninfected (HUU) infants at birth and 6 months. **(E,F)** The mean fluorescence intensity of IFNγ^+^ NK cells is compared between HEU and HUU infants at birth and 6 months. Mann–Whitney tests were used to compare groups, and *p* values are shown where *p* < 0.05. Error bars indicate median and interquartile range.

Activated NK cells produce and secrete the cytokine IFNγ. We used intracellular staining to compare NK cell IFNγ production by stimulated HEU and HUU NK cells. We observed that fewer birth HEU NK cells produced IFNγ following stimulation with K562 targets [0.77% (−4.02 to 2.51) vs. 2.64% (0.94–5.56), *p* = 0.008 (Figures 3A,B)]. The proportion of HEU NK cells producing IFNγ was higher at 6 months of age but still lower than for HUU NK cells [4.12% (3.18–5.68) vs. 8.39% (5.9–9.98), *p* = 0.001 (Figures 3C,D)]. When the MFI of IFNγ staining was compared in the respective IFNγ^+^ NK cell populations, cytokine production was also significantly lower at both birth and 6 months in the HEU NK cells [birth: 796 (634–1,206) vs. 1511 (1,298–1,812), *p* = 0.0002; 6 months: 1926 (1,252–2,116) vs. 2337 (2,046–2,539), *p* = 0.0003 (Figures 3E,F)]. The reduced IFNγ production by HEU NK cells was observed in both CD16^+^ and CD16^−^ NK cell subsets.

**Figure 4:**
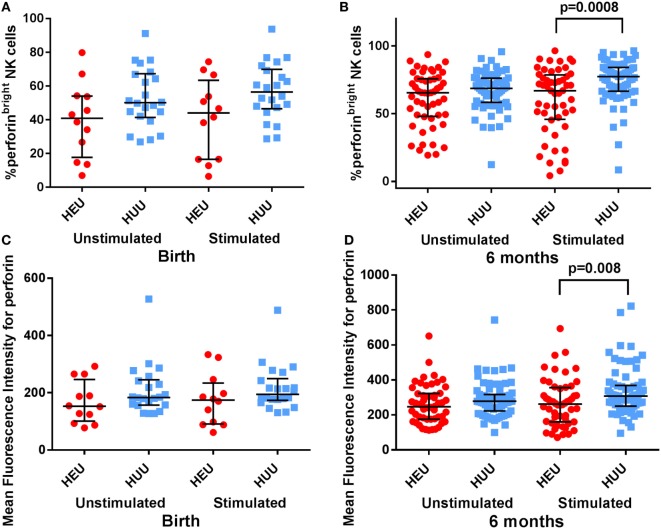
**Perforin expression by natural killer (NK) cells**. **(A,B)** The proportion of perforin^bright^ NK cells is compared between HIV-exposed uninfected (HEU) and HIV-unexposed uninfected (HUU) infants at birth and 6 months. **(C,D)** The mean fluorescence intensity of perforin on all NK cells is compared between HEU and HUU infants at birth and 6 months. Mann–Whitney tests were used to compare groups, and *p* values are shown where *p* < 0.05. Error bars indicate median and interquartile range.

Activated NK cells also increase their production of granules and granule-associated proteins such as perforin. Following stimulation with K562 targets, we found that the proportion of perforin^bright^ NK cells was lower in HEU vs. HUU infants both at birth and at 6 months, albeit this difference was only significant at 6 months [birth: 44.05% (15.60–66.60) vs. 56.45% (47.20–69.20), *p* = 0.08; 6 months: 66.95% (55.30–73.10) vs. 77.30% (71.10–80.30), *p* = 0.0008 (Figures 4A,B)]. The MFI for perforin was also lower in stimulated HEU vs. HUU NK cells at 6 months [262 (212–293) vs. 308 (274–327); *p* = 0.008 (Figures 4C,D)]. Perforin expression was higher in the CD16^+^ compared with CD16^−^ NK cell subsets for both groups, but differences between HEU and HUU perforin expression were primarily seen in the CD16^−^ NK cell subset.

### NK Cell-Activating and Inflammatory Cytokines in K562-Stimulated Cultures of HEU and HUU Infants

**Figure 5:**
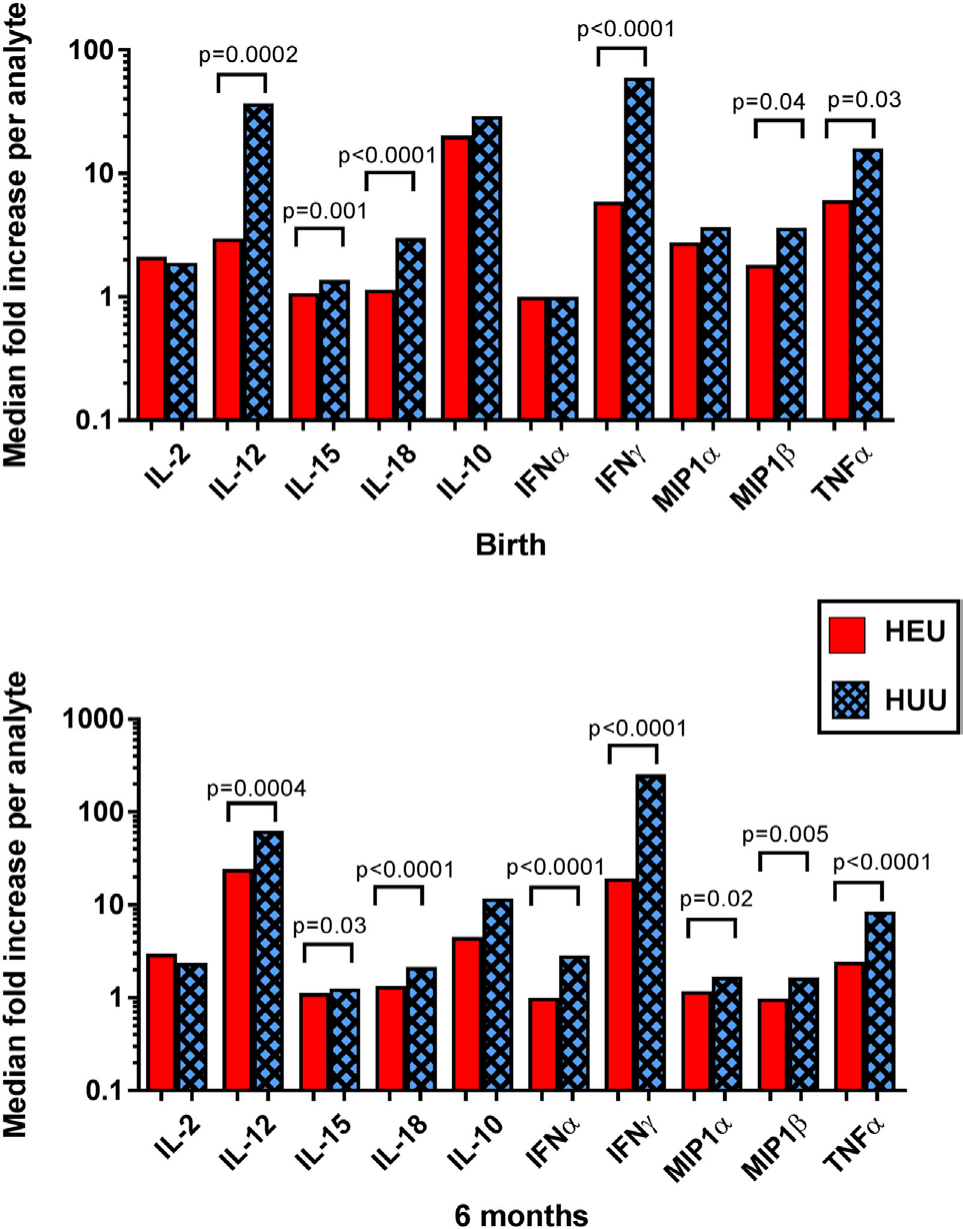
**Detection of cytokines and chemokines in cell culture supernatant**. The median fold increase in analyte detection between unstimulated and stimulated cell culture supernatants is shown for each cytokine or chemokine at birth and 6 months. Mann–Whitney tests were used to compare HIV-exposed uninfected (HEU) and HIV-unexposed uninfected (HUU) groups. *p* values are shown where *p* < 0.05.

Evaluation of supernatant from K562-stimulated HEU cell cultures at birth and 6 months revealed lower levels of cytokines previously implicated in NK activation, including IL-12, IL-15, and IL-18, compared with HUU cell cultures (Figure 5). The greatest difference between HEU and HUU was observed for IL-12 [birth: median fold increase of 2.97 (1.00–8.39) vs. 36.97 (6.49–66.83), *p* = 0.0002; 6 months: 24.50 (7.88–42.22) vs. 62.70 (34.88–116.30), *p* = 0.0004]. HEU cell culture supernatants also demonstrated significantly fewer inflammatory cytokines compared with HUU, including IFNα (6 months only), IFNγ, MIP1α (6 months only), MIP1β, and TNFα (Figure 5). Of note, when K562 cells were cultured alone they produced a small amount of IL-15 (range 1.3–2.2 pg/mL) and IL-18 (100.4–131.7 pg/mL) but did not produce any other tested cytokine or chemokine.

### Effect of Reconstitution with Exogenous rhIL-12 on NK Cell Production of IFNγ

**Figure 6:**
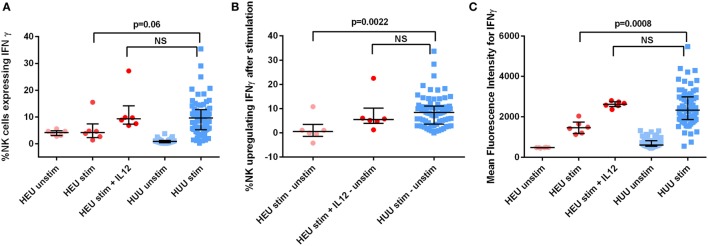
**HIV-exposed uninfected (HEU) natural killer (NK) cell production of interferon (IFN)γ after effect reconstitution with exogenous rhIL-12**. Six HEU 6-month samples were stimulated with and without rhIL-12 at a concentration of 0.1 ng/mL. **(A)** The proportion of NK cells expressing IFNγ, **(B)** the proportion of NK cells upregulating IFNγ (defined as proportion of stimulated NK cells expressing IFNγ—proportion of unstimulated NK cells expressing IFNγ), and **(C)** the mean fluorescence intensity of IFNγ^+^ NK cells is compared between HEU and HIV-unexposed uninfected (HUU) infants at 6 months of age. Mann–Whitney tests were used to compare groups. *p* values are shown for each comparison of HEU and HUU NK cells stimulated without rhIL-12. NS, not significant. Error bars indicate median and interquartile range.

We assessed the effect of reconstitution with exogenous recombinant human (rh)IL-12 on HEU NK cell production of IFNγ. IL-12 was chosen for these experiments because the differences in IL-12 concentration between HEU and HUU cell culture supernatants were greater than the differences in concentrations of other cytokines known to trigger NK cell activation. We incubated six HEU 6-month samples with K562 cells and exogenous rhIL-12 at a concentration of 0.1 ng/mL and compared with HUU samples stimulated without exogenous rhIL-12. The addition of IL-12 resulted in IFNγ production by HEU NK cells comparable to that of K562-stimulated HUU NK cells without exogenous rhIL-12. The proportion of HEU NK cells that upregulated IFNγ [5.48% (1.25–22.53) vs. 8.39% (5.79–9.98); *p* = 0.41] and the MFI of IFNγ^+^ HEU NK cells stimulated in the presence of exogenous rhIL-12 [2,623 (2,360–2,803) vs. 2,337 (2,046–2,539); *p* = 0.30] were not significantly different from those of HUU NK cells stimulated without exogenous rhIL-12 (Figure 6). These data suggest that impaired cytokine production by accessory immune cells may contribute to the observed decreases in HEU NK cell function and show that HEU NK cells are capable of more robust functional responses if given appropriate environmental signals.

### Risk of LRTI According to NK Cell Phenotype and Function

Of the 12 HEU infants whose birth samples were studied, 6 developed LRTI in the first 6 months of life. When LRTI^+^ HEU infants’ NK cell phenotype and function were compared with LRTI^−^ HEU infants, there were trends toward decreased killing of target cells at birth (*p* = 0.056; Figure S4 in Supplementary Material) and decreased production of perforin (*p* = 0.065), but no statistically significant differences.

## Discussion

In these cohorts of HEU and HUU infants from Brazil, we demonstrated differences in NK cell proportion, phenotype, and function. In HEU infants, the proportions of NK cells were lower than in HUU infants at birth and 6 months. HUU infants demonstrated a high proportion of NK cells at birth that declined by 6 months of age. Consistent with our observations in the HUU infants, previous studies have found that the proportion of NK cells in healthy infant blood typically peaks at birth and falls to adult levels by 5 years of age (33, 34). A prior study of HEU adolescents found reduced proportions of NK cells in peripheral blood compared with healthy controls (35). Thus, the NK cell defect we observed in HEU infants may persist as they age. It is not clear why NK cell numbers are reduced in this population. One possibility is that NK cells in the HEU infants are more susceptible to apoptosis, as described for T- and B-cells in this population (36, 37). However, Slyker et al. failed to observe increased apoptosis susceptibility in cultured NK cells from HEU compared with HUU infants (26). Reduced NK cell numbers might also reflect reduced production or maintenance of NK cell precursors in the HEU infants, or depletion of these precursors *in utero*. Finally, some studies have suggested that formula feeding, which occurred more frequently in the HEU infants, can also contribute to lower NK cell frequencies in peripheral blood (38).

Despite the smaller proportion of NK cells in HEU infants, we found that the ability of these NK cells to degranulate and induce cytotoxicity was preserved or even enhanced compared with HUU infants. We propose that the increased cytotoxicity of NK cells from HEU infants is attributable to increased cell activation, which in turn could be triggered by *in utero* exposure to inflammatory stimuli. This hypothesis is consistent with the increased activation markers described in other lymphocyte subsets from HEU infants (39–41). While we did not specifically examine activation markers on NK cells, Slyker et al. reported increased proportions of early activated (CD38^+^CD69^+^) NK cells in HEU compared with HUU infants (26). In that study, the majority of early activated NK cells were in the CD56^dim^ subset, which is thought to be primarily responsible for cytotoxicity (33). We found increased CD107a expression in CD16^+^ NK cells from HEU infants, which supports the hypothesis that early activation in this subset could be responsible for increased cytotoxicity. We did not find any differences in the expression of NKG2C, a marker of activation associated with viral infections, between HEU and HUU infants. However, deletion of the NKG2C gene has been reported with varying prevalence in different populations (42); this may have limited our ability to detect differences in the expression of this marker.

Despite their increased cytotoxicity, we found that fewer HEU NK cells express IFNγ and perforin than NK cells from HUU infants. Slyker et al. also reported a reduction in perforin^+^ NK cells over the first few months of life in HEU infants, but an increase in HUU infants (26). Low perforin expression could reflect increased degranulation of more highly activated NK cells. However, expression of IFNγ was also reduced in HEU infants’ NK cells. We thus favor the model that HEU NK cells synthesize less of these factors, presumably because they receive less stimulation by activating cytokines. Indeed, we observed reduced IL-12, IL-15, and IL-18 in HEU cell cultures. The largest difference in cytokine concentrations was noted for IL-12. Poor IL-12 production by HEU infants’ cord blood mononuclear cells and PBMCs has been previously described (43). We further found that reconstituting the IL-12 concentrations with exogenous rhIL-12 in HEU cell cultures resulted in improved IFNγ production by HEU NK cells, which approached levels similar to HUU NK cells. We reconstituted IL-12 using the manufacturer-recommended concentration of 0.1 ng/mL of exogenous rhIL-12, which was within the range (0.0001–0.40 ng/mL) of IL-12 concentrations detected in stimulated HUU cell culture supernatants, but higher than the median concentration of 0.012 ng/mL.

The improved IFNγ expression after IL-12 reconstitution suggests that the deficient NK cell responses found in HEU infants are at least partially due to insufficient environmental stimuli necessary to induce NK cell maturity and function. IL-12, IL-15, and IL-18 are typically produced by antigen-presenting cells (APCs) such as dendritic cells and monocytes. We found that K562 cells produced a small amount of IL-15 and IL-18, but did not produce any IL-12, indicating that IL-12 in our cultures was likely attributable to production by infant APCs. In our study, the only stimulant provided in cell cultures was the K562 cells. K562 cells are thought to activate NK cells primarily through the missing-self mechanism, due to their lack of a major histocompatibility complex. K562 cells do not express co-stimulatory molecules such as CD83 and CD86, and they are not known to directly stimulate APCs (44). It is unclear how the APCs are stimulated to produce IL-12 in our experiments. This is not likely due to contamination of the K562 cells with pathogen-associated molecular patterns (PAMPs) since a previous study found that APCs from 2-week-old HEU infants produce higher amounts of IL-12 than HUU APCs after stimulation with PAMPs (45). Perhaps, the activated NK cells themselves stimulate IL-12 production in APCs. Hence, decreased cytokine production by APCs in our cell cultures might result from inadequate signaling from NK cells to APCs or a defect intrinsic to the APCs. Regardless, the low functionality of the NK cells in HEU infants is likely to derive from the interactions between NK cells and APCs. Further studies are warranted to better define how cellular cross-talk between NK cells and APCs is altered in HEU infants.

We attempted to associate NK cell characteristics in HEU infants with risk of LRTI in the first 6 months of life. However, our sample size was too small to detect significant differences in any of the NK cell parameters. NK cells are thought to be important for the immune response to several respiratory viral infections, including influenza and RSV (22, 23, 46). In addition, NK cell defects might be a marker of global immune dysfunction in HEU infants, which could predispose them to an increased risk of LRTI. Larger studies are needed to determine if the altered NK cell function and phenotype at birth in HEU infants may predict increased risk of LRTI in the first 6 months of life.

Strengths of our study include that it is the largest evaluation of NK cells in HEU infants to date, and the first to evaluate NK cell function in HEU infants. Similar to other immunology studies that have been performed in HEU infants, it is not possible to distinguish whether the NK cell perturbations that we identified result from exposure to HIV itself versus exposure to drugs, maternal co-infections, or other factors associated with maternal HIV infection. We used a HUU infant control group that was relatively well matched for location, ethnicity, and socioeconomic status, thus minimizing any differences attributable to genetic or environmental factors. However, HEU and HUU infants were enrolled during different years and differed in their exposure to breast milk. We were unable to assess for differences in HEU infants’ NK cell proportion, phenotype, or function according to the type of antiretroviral drugs administered to mothers during pregnancy, although antiretroviral exposure is unlikely to have influenced infant NK cell parameters. Additional weaknesses include our inability to assess for additional phenotypic changes, such as the expression of NK cell activation markers, given the limitations of our staining panel. Our comparison of infants within each cohort at birth vs. 6 months was limited because very few infants were followed longitudinally. In addition, the number of infants included in some of our analyses, especially the comparison of LRTI^+^ vs. LRTI^−^ HEU infants, was too small to detect significant differences. Therefore, our findings should be confirmed with a prospective study that enrolls large numbers of HEU and HUU infants.

## Conclusion

The pathogenesis of increased morbidity and mortality in HEU infants is unclear but appears to be related to immune deficiency that occurs as a result of some *in utero* exposure. This study contributes to the understanding of the innate immune system of HEU infants by showing distinct differences in NK cell proportion, phenotype, and function compared with HUU infants. NK cells in HEU infants appear to be affected by perinatal exposure to HIV, either directly or as a result of impaired signaling from other cell types. Further investigation into the behavior of the HEU infant’s immune system is important for the development of interventions that can decrease their risk of infectious morbidity and mortality.

## Ethics Statement

This study was carried out in accordance with the recommendations of the Colorado Multiple Institutional Review Board, Westat Institutional Review Board, the Brazil National Commission of Ethics in Research and Institution Ethics Committee/IRB (CONEP), and Comitê de Ética em Pesquisa do HCRP e da FMRP-USP. Written informed consent, including permission to use biological materials, was obtained from all subjects. All subjects gave written informed consent in accordance with the Declaration of Helsinki. The protocol was approved by the Colorado Multiple Institutional Review Board, Westat Institutional Review Board, the Brazil National Commission of Ethics in Research and Institution Ethics Committee/IRB (CONEP), and Comitê de Ética em Pesquisa do HCRP e da FMRP-USP.

## Author Contributions

CS, EJ, VA, JC, LL, MM-P, FA, JP, JA, GS, and AW contributed to the conception and design of the work. CS, EJ, VA, JC, and AW contributed to data collection. CS, EJ, LL, RC, QY, and AW contributed to the data analysis and interpretation. CS drafted the manuscript. EJ, VA, JC, LL, MM-P, RC, QY, FA, JP, JA, GS, and AW contributed to the critical revision of the manuscript. CS, EJ, VA, JC, LL, MM-P, RC, QY, FA, JP, JA, GS, and AW approved the final version of the manuscript to be published and agreed to be held accountable for all aspects of the work.

## Conflict of Interest Statement

AW receives research funds from MedImmune, Merck, and GlaxoSmithKline. The remaining authors declare that the research was conducted in the absence of any commercial or financial relationships that could be construed as a potential conflict of interest. The reviewer MB and handling Editor declared their shared affiliation, and the handling Editor states that the process nevertheless met the standards of a fair and objective review.

## Appendix of Additional Study Sites and Participants

Principal investigators, *co-principal investigators*, study coordinators, coordinating center representatives, and NICHD staff include:

Marcelo H. Losso, Hospital General de Agudos José María Ramos Mejía, Buenos Aires, Argentina; Irene Foradori, Hospital General de Agudos José María Ramos Mejía, Buenos Aires, Argentina; Alejandro Hakim, Hospital General de Agudos José María Ramos Mejía, Buenos Aires, Argentina; Erica Stankievich, Hospital General de Agudos José María Ramos Mejía, Buenos Aires, Argentina; Silvina Ivalo, Hospital General de Agudos José María Ramos Mejía, Buenos Aires, Argentina; Jorge A. Pinto, Universidade Federal de Minas Gerais, Belo Horizonte, Brazil; *Victor H. Melo*, Universidade Federal de Minas Gerais, Belo Horizonte, Brazil; Fabiana Kakehasi, Universidade Federal de Minas Gerais, Belo Horizonte, Brazil; Beatriz M. Andrade, Universidade Federal de Minas Gerais, Belo Horizonte, Brazil; Rosa Dea Sperhacke, Universidade de Caxias do Sul, Serviço Municipal de Infectologia, Caxias do Sul, Brazil; *Nicole Golin*, Universidade de Caxias do Sul, Serviço Municipal de Infectologia, Caxias do Sul, Brazil; Sílvia Mariani Costamilan, Universidade de Caxias do Sul, Serviço Municipal de Infectologia, Caxias do Sul, Brazil; Jose Pilotto, Hospital Geral Nova de Iguacu—HIV Family Care Clinic, Nova Iguacu, Brazil; *Luis Eduardo Fernandes*, Hospital Geral Nova de Iguacu—HIV Family Care Clinic, Nova Iguacu, Brazil; Gisely Falco, Hospital Geral Nova de Iguacu—HIV Family Care Clinic, Nova Iguacu, Brazil; Rosa Dea Sperhacke, Universidade de Caxias do Sul, Hospital Conceição, Porto Alegre, Brazil; *Breno Riegel Santos*, Universidade de Caxias do Sul, Hospital Conceição, Porto Alegre, Brazil; Rita de Cassia Alves Lira, Universidade de Caxias do Sul, Hospital Conceição, Porto Alegre, Brazil; Rosa Dea Sperhacke, Universidade de Caxias do Sul, Hospital Fêmina, Porto Alegre, Brazil; *Mario Ferreira Peixoto*, Universidade de Caxias do Sul, Hospital Fêmina, Porto Alegre, Brazil; Elizabete Teles, Universidade de Caxias do Sul, Hospital Fêmina, Porto Alegre, Brazil; Regis Kreitchmann, Irmandade da Santa Casa de Misericordia de Porto Alegre, Porto Alegre, Brazil; *Luis Carlos Ribeiro*, Irmandade da Santa Casa de Misericordia de Porto Alegre, Porto Alegre, Brazil; *Fabrizio Motta*, Irmandade da Santa Casa de Misericordia de Porto Alegre, Porto Alegre, Brazil; Debora Fernandes Coelho, Irmandade da Santa Casa de Misericordia de Porto Alegre, Porto Alegre, Brazil; Marisa M. Mussi-Pinhata, Hospital das Clínicas da Faculdade de Medicina de Ribeirão Preto da Universidade de São Paulo, Ribeirão Preto, Brazil; *Geraldo Duarte*, Hospital das Clínicas da Faculdade de Medicina de Ribeirão Preto da Universidade de São Paulo, Ribeirão Preto, Brazil; Adriana A. Tiraboschi Bárbaro, Hospital das Clínicas da Faculdade de Medicina de Ribeirão Preto da Universidade de São Paulo, Ribeirão Preto, Brazil; Conrado Milani Coutinho, Hospital das Clínicas da Faculdade de Medicina de Ribeirão Preto da Universidade de São Paulo, Ribeirão Preto, Brazil; Fabiana Rezende Amaral, Hospital das Clínicas da Faculdade de Medicina de Ribeirão Preto da Universidade de São Paulo, Ribeirão Preto, Brazil; Anderson Sanches de Melo, Hospital das Clínicas da Faculdade de Medicina de Ribeirão Preto da Universidade de São Paulo, Ribeirão Preto, Brazil; Ricardo Hugo S. Oliveira, Instituto de Puericultura e Pediatria Martagão Gesteira, Rio de Janeiro, Brazil; *Elizabeth S. Machado*, Instituto de Puericultura e Pediatria Martagão Gesteira, Rio de Janeiro, Brazil; Maria C. Chermont Sapia, Instituto de Puericultura e Pediatria Martagão Gesteira, Rio de Janeiro, Brazil; Esau Custodio Joao, Hospital dos Servidores do Estado, Rio de Janeiro, Brazil; Leon Claude Sidi, Hospital dos Servidores do Estado, Rio de Janeiro, Brazil; Maria Leticia Santos Cruz, Hospital dos Servidores do Estado, Rio de Janeiro, Brazil; Maria Isabel Gouvêa, Hospital dos Servidores do Estado, Rio de Janeiro, Brazil; Mariza Curto Saavedra, Hospital dos Servidores do Estado, Rio de Janeiro, Brazil; Clarisse Bressan, Hospital dos Servidores do Estado, Rio de Janeiro, Brazil; Fernanda Cavalcanti A. Jundi, Hospital dos Servidores do Estado, Rio de Janeiro, Brazil; Regina Celia de Menezes Succi, Escola Paulista de Medicina, Universidade Federal de São Paulo, São Paulo, Brazil; Prescilla Chow, Escola Paulista de Medicina, Universidade Federal de São Paulo, São Paulo, Brazil; Jorge O. Alarcón Villaverde, Instituto de Medicina Tropical “Daniel Alcides Carrión”, Sección de Epidemiología, UNMSM, Lima, Peru; *Carlos Velásquez Vásquez*, Instituto Nacional Materno Perinatal, Lima, Peru; César Gutiérrez Villafuerte Instituto de Medicina Tropical “Daniel Alcides Carrión”, Sección de Epidemiología, UNMSM, Lima, Peru; Yolanda Bertucci, Data Management and Statistical Center, Westat, Rockville, MD, USA; Rachel Cohen, Data Management and Statistical Center, Westat, Rockville, MD, USA; Laura Freimanis Hance, Data Management and Statistical Center, Westat, Rockville, MD, USA; René Gonin, Data Management and Statistical Center, Westat, Rockville, MD, USA; D. Robert Harris, Data Management and Statistical Center, Westat, Rockville, MD, USA; Roslyn Hennessey, Data Management and Statistical Center, Westat, Rockville, MD, USA; James Korelitz, Data Management and Statistical Center, Westat, Rockville, MD, USA; Margot Krauss, Data Management and Statistical Center, Westat, Rockville, MD, USA; Sue Li, Data Management and Statistical Center, Westat, Rockville, MD, USA; Karen Megazzini, Data Management and Statistical Center, Westat, Rockville, MD, USA; Orlando Ortega, Data Management and Statistical Center, Westat, Rockville, MD, USA; Sharon Sothern de Sanchez, Data Management and Statistical Center, Westat, Rockville, MD, USA; Sonia K. Stoszek, Qilu Yu, Westat, Rockville, MD, USA; George K. Siberry, NICHD, *Eunice Kennedy Shriver* National Institute of Child Health and Human Development, Bethesda, MD, USA; Rohan Hazra, NICHD, *Eunice Kennedy Shriver* National Institute of Child Health and Human Development, Bethesda, MD, USA; Lynne M. Mofenson, NICHD, *Eunice Kennedy Shriver* National Institute of Child Health and Human Development, Bethesda, MD, USA.

## Abbreviations

APCs, antigen-presenting cells; HEU, HIV-exposed uninfected; HUU, HIV-unexposed uninfected; IFN, interferon; IL, interleukin; LRTI, lower respiratory tract infection; MFI, mean fluorescence intensity; NK, natural killer; PAMP, pathogen-associated molecular pattern; PBMCs, peripheral blood mononuclear cells; RSV, respiratory syncytial virus.
