# Supplementary material for: Altered Natural Killer Cell Function in HIV-Exposed Uninfected Infants
Source: Front Immunol. 2017 Apr 24;8:470. doi: 10.3389/fimmu.2017.00470 (PMC5403425; doi:10.3389/fimmu.2017.00470)
Supplement: Supplementary file 1 [file data_sheet_1.docx]

Supplementary Material

**Altered Natural Killer Cell Function in HIV-Exposed Uninfected Infants**

**Christiana Smith^1*^, Emilie Jalbert^1^, Volia de Almeida^2^, Jennifer Canniff^1^, Laurel L. Lenz^3^, Marisa M. Mussi-Pinhata^4^, Rachel A. Cohen^5^, Qilu Yu^5^, Fabiana R. Amaral^4^, Jorge Pinto^6^, Jorge O. Alarcon^7^, George Siberry^8^, and Adriana Weinberg^1^, for the NISDI LILAC and CIRAI study teams**

***Correspondence:**

Christiana Smith

[Christiana.Smith@childrenscolorado.org](mailto:Christiana.Smith@childrenscolorado.org)

1. **Supplementary Figures and Tables**

**1.1 Supplementary Figures**

1630 HIV-infected pregnant women enrolled in the NISDI perinatal and LILAC studies

675 mother-infant pairs met inclusion criteria

288 mother-infant pairs met matching criteria

387 mother-infant pairs did not have an appropriate match

144 LRTI+

144 LRTI-

**119 LRTI+**

25 had LRTI after 6 months of life: shifted to LRTI-

169 LRTI-

**161 LRTI-**

8 LRTI- without LRTI+ match

**N=247 mother-infant pairs**

21 infants excluded due to pneumococcal vaccine

12 infants excluded due to pneumococcal vaccine

955 mothers and/or their infants did not meet inclusion criteria

**Supplemental Figure 1.** Flow diagram depicting selection of HEU infants.

106 HIV-uninfected pregnant women and their infants met inclusion criteria

**N=88 mother-infant pairs**

18 mother-infant pairs excluded due to change in breastfeeding status or blood sample not collected

**Supplemental Figure 2.** Flow diagram depicting selection of HUU infants.

**
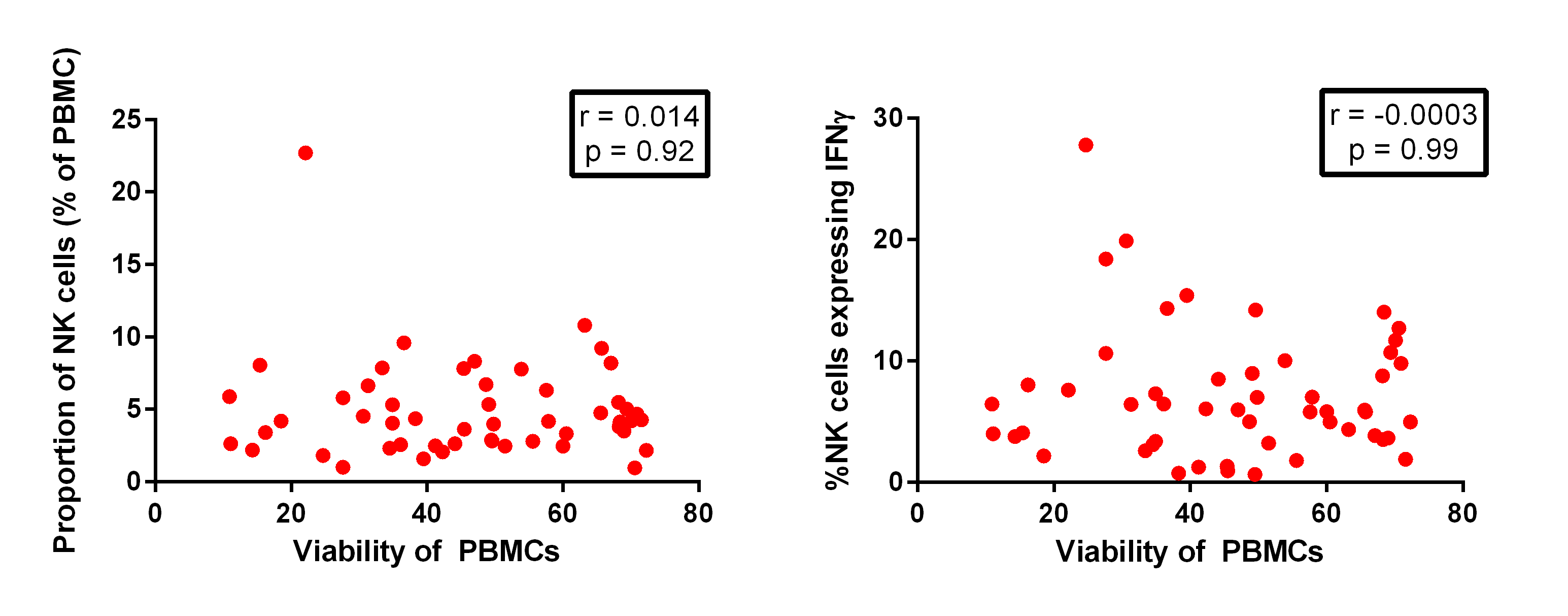
**

**Supplemental Figure 3.** Correlation analyses showed no association between the viability of peripheral blood mononuclear cells (PBMCs) after stimulation and the proportion of natural killer cells among PBMCs, or the percentage of NK cells that expressed IFNγ after stimulation.


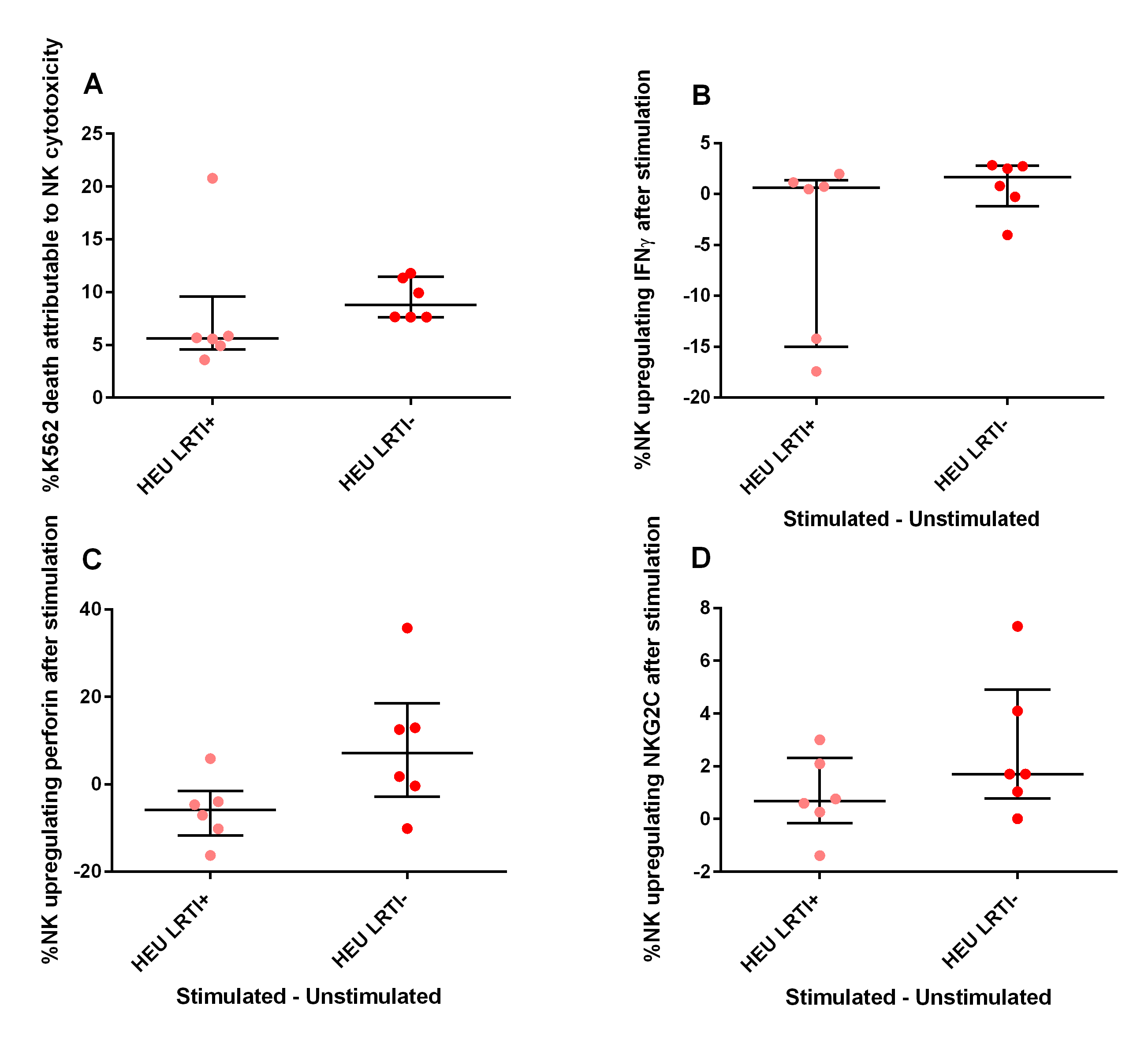


**Supplemental Figure 4. Comparison of six HEU infants who did (LRTI^+^) and six who did not (LRTI^-^) develop lower respiratory tract infection in the first six months of life.** (A) The proportion of K562 death attributable to NK cytotoxicity is compared between LRTI^+^ and LRTI^-^ HEU infants at birth. The proportion of NK cells upregulating (B) IFNγ, (C) perforin, and (D) NKG2C, defined as proportion of stimulated NK cells expressing marker – proportion of unstimulated NK cells expressing marker), is compared between LRTI^+^ and LRTI^-^ HEU infants at birth. Mann-Whitney tests were used to compare groups; there were no significant differences. Error bars indicate median and interquartile range.

**1.2 Supplementary Tables**

**Supplemental Table 1.** Demographic characteristics of mothers and infants in entire cohort according to infant HIV exposure category.

| **Maternal Characteristics** | **Infant HIV exposure category** | | **p-value^a^** |
| --- | --- | --- | --- |
|  | **HEU**  (N = 247) | **HUU**  (N=88) |  |
| Mean (SD) maternal age at enrollment | 26.9 (5.5) | 25.7 (5.8) | 0.08 |
| Substance use during pregnancy, N (%)  Alcohol  Tobacco  Marijuana  Crack or cocaine | 49 (20.0)  58 (23.8)  9 (3.8)  8 (3.4) | 9 (10.2)  12 (13.6)  0 (0.0)  0 (0.0) | 0.05  0.05  0.12  0.11 |
| Maternal employment, N (%) | 73 (29.6) | 50 (56.8) | <0.0001 |
| Mean (SD) years of completed education | 7.4 (3.2) | 9.3 (2.4) | <0.0001 |
| Mean (SD) number of prior pregnancies | 2.3 (2.1) | 1.1 (1.2) | <0.0001 |
| Mean (SD) parity | 1.7 (1.6) | 0.9 (1.1) | <0.0001 |
| Mean (SD) number of people living in the household | 4.2 (2.5) | 4.5 (1.7) | 0.11 |
| Mean (SD) CD4 count (cells/mm^3^) at hospital discharge | 558.7 (270.7) | - | - |
| Mean (SD) CD4% at hospital discharge | 31.7 (10.5) | - | - |
| Mean (SD) viral load (log_10_ copies/mL) at hospital discharge | 2.62 (0.81) | - | - |
| Viral load <400 copies/mL at hospital discharge, N (%) | 80 (48.2) | - | - |
| ARV use during pregnancy, N (%)  Zidovudine monotherapy  Combination antiretrovirals  Unknown | 127 (51.4)  119 (48.2)  1 (0.4) | -  -  - | -  -  - |
| **Infant Characteristics** | | | |
| Mean (SD) gestational age at birth (weeks) | 38.7 (1.1) | 39.3 (1.4) | 0.001 |
| Female gender, N (%) | 100 (40.5) | 43 (48.9) | 0.21 |
| Mean (SD) birth weight (grams) | 3159.2 (378.7) | 3382.2 (390.9) | <0.0001 |
| Small for gestational age (<10^th^ percentile), N (%) | 8 (3.2) | 0 (0.0) | 0.12 |
| Large for gestational age (>90^th^ percentile), N (%) | 27 (10.9) | 22 (25.0) | 0.003 |
| Not breastfeeding, N (%) | 247 (100) | 1 (1.1) | <0.0001 |

^a^All p-values were calculated by two-sample t-test or chi-square/Fischer’s exact test, where appropriate.

Note: Maternal race was not analyzed due to the inability to establish it in this highly mixed Brazilian population.

ARV, antiretroviral; HEU, HIV-exposed uninfected infant; HUU, HIV-unexposed uninfected infant; N, number; SD, standard deviation
